# Evaluation of the utility of testicular-only processing fluid for porcine reproductive and respiratory syndrome virus diagnostics and the effect of sample pooling on the test results

**DOI:** 10.2478/jvetres-2025-0011

**Published:** 2025-03-01

**Authors:** Hanna Turlewicz-Podbielska, Arkadiusz Dors, Małgorzata Pomorska-Mól

**Affiliations:** Department of Preclinical Sciences and Infectious Diseases, Faculty of Veterinary Medicine and Animal Sciences, Poznań University of Life Sciences, 60-637 Poznań, Poland

**Keywords:** diagnostic matrices, porcine reproductive and respiratory syndrome, processing fluid, testicular-only processing fluid, pigs

## Abstract

**Introduction:**

The testicular-only processing fluid (TOPF) obtained from piglet testicles after castration could be an alternative sample for porcine reproductive and respiratory syndrome (PRRS) laboratory diagnosis. If this matrix were proved useful, testing it would spare piglets the stress of blood drawing and eliminate some labour required to take blood samples. The aim of the study was to evaluate the utility of TOPF for this diagnostic purpose.

**Material and Methods:**

Serum-and-TOPF pairs from male piglets and sera from female piglets were tested using commercial ELISA and real-time RT-PCR kits. For the pooling simulation, 10 μL aliquots of TOPF separated into low-, moderately and highly positive were mixed with appropriate volumes of negative TOPF samples. This simulated pools of 5, 10, 20, 40 and 80 samples containing 1 positive for serological analyses and pools of 10, 20, 40, 80, 160 and 320 samples containing 1 positive in molecular analyses.

**Results:**

The percentages of anti-porcine reproductive and respiratory syndrome virus (PRRSV) antibodies were statistically significantly different (P-value < 0.05) between boar sera (69.55%) and TOPF (54.49%), as well as between gilt sera (74.52%) and TOPF. However, after adjusting the cut-off value, no significant differences were noted. The RNA of PRRSV was detected in 21.26% of male sera, 15.23% of TOPFs and 17.00% of female sera. Pooled sample testing revealed discrepancies in positive results associated with the pool size and original sample positivity strength.

**Conclusion:**

TOPF samples can be a valuable matrix for laboratory PRRS diagnosis in piglets. However, it is important to be aware of the potential for false-negative results.

## Introduction

Porcine reproductive and respiratory syndrome (PRRS) is a viral infectious disease that has a detrimental impact on the pig farming industry globally, causing significant economic losses (2, 19). The syndrome inflicts severe damage on pigs at various stages of growth (2). In the USA, PRRS outbreaks led to estimated annual financial losses of around $560 million in 2005. By 2013, productivity losses in the USA due to PRRS were calculated to be as high as $664 million per year (13, 20). A recent study in Germany focused on the economic impact of PRRS virus (PRRSV) in the country found that all 21 investigated farms experienced significant losses attributable to the pathogen. The median total loss per farm per year due to PRRS was €74,181, corresponding to a median total loss per sow and year of €255. On average, the impact of PRRS on farm profits was –19.1%, and in the worst case, it was –41% (23). Porcine reproductive and respiratory syndrome virus requires sustained and widespread attention as a globally important swine pathogen (7).

The genome of PRRSV has revealed increasing genetic differences over the years, leading to the classification of the virus into two distinct species: *Betaarterivirus suid 1* (previously known as the European type or PRRSV-1) and *Betaarterivirus suid 2* (previously known as the American type or PRRSV-2) (3). The syndrome caused by *Betaarterivirus suid 1* and *Betaarterivirus suid 2* is recognised for causing respiratory symptoms, reduced average daily weight gain, mortality in growing/fattening pigs and reproductive problems in sows. The severity of the disease’s clinical signs and financial impact varies significantly depending on viral species and strain, host immune status and age, co-infections, and disease stage. The disease can also be subclinical (10, 30).

The use of non-invasive sampling or the use of material that would usually be discarded (*e.g*., testicles after castration) for diagnostic purposes can help not only reduce the stress of blood collection, which is the regular *ex vivo* diagnostic material from pigs, but also increase the frequency of diagnostic testing in the herd. Early diagnosis is crucial for effective control of infection in swine herds. Using samples obtained during routine procedures implemented on the farm can significantly save time, labour and costs, improving production profitability. Minimising the stress associated with blood collection is also crucial for animal welfare. Therefore, using non-invasive matrices that could replace traditional samples is essential, and should be standard policy for detecting economically important pathogens commonly found on farms (26).

Processing fluid (PF) shows promise for monitoring viral diseases in breeding herds and suckling piglets. A serosanguinous exudate obtained during castration and tail-docking of piglets, PF can be put to good use for detecting PRRSV. Processing fluid has been previously demonstrated to provide a practical and sensitive matrix for PRRSV genetic material detection in viraemic piglets (14–17, 25, 27, 28). In pig farming, there are certain management practices that are carried out during the first week of life, such as piglet processing (including castration and tail docking). Swine castration is performed to control aggressive behaviour and improve pork taste by removing most boar taint. Tail docking is primarily carried out to prevent cannibalism. These practices facilitate the collection of samples which can be used to monitor the epidemiological status of the herd without causing significant inconvenience (27). However, European legislation dictates that tail docking should not be carried out routinely (Council Directive 2008/120/EC of December 18 2008, laying down minimum standards for the protection of pigs); therefore, in our research, we decided to use a model in which the PF consisted only of fluid from testes: testicular-only processing fluid (TOPF).

This study aimed to assess the utility of TOPF collected during piglet castration for PRRSV serological and molecular laboratory diagnosis and the effect of sample pooling on the test results. In addition, whether results obtained from TOPF differed from results obtained from serum collected from male and female piglets was investigated.

## Material and Methods

### Farms

The study was conducted on 18 Polish commercial pig farms where surgical castration and serological health status monitoring were routinely performed. Two herds had previously been diagnosed as PRRSV-positive, and the status of the remaining farm was unknown.

### Sample collection

Samples were collected from 697 3–5-day-old piglets from 62 litters, from 1 to 15 litters per herd. Not all samples were subjected to both molecular and serological analyses because the amount of blood available was limited. Blood samples were collected from the *vena cava cranialis* into clot activator tubes. Testes from individual male piglets were collected into 50 mL Falcon Tubes (Fisher Scientific, Hampton, NH, USA). Tail docking was not always performed on the farms where the samples were collected. Besides from male piglets, blood samples were also collected from the 263 female piglets which were littermates of the sampled males. All samples were transported to a laboratory in packaging with cooling inserts. Blood was centrifuged at 2,500 × *g* for 15 min at 4°C to obtain serum. To obtain TOPF, the testes were frozen and thawed twice. Serum and TOPF were kept frozen at –80° until further analysis.

All samples used in the present study were taken for diagnostic purposes by the veterinarian in charge of the herd and during routine castrations. Therefore, no ethical approval was necessary. Individual oral informed consent was obtained from the owners for the participation of their animals in this study.

### Detection of antibodies against PRRSV and pooling simulation

A total of 312 serum-and-TOPF pairs from male piglets and sera from 263 female piglets were tested serologically. The presence of PRRSV-specific immunoglobulin G antibodies (Ab) in the serum and TOPF were evaluated using a commercial ELISA kit (PRRS Ab 4.0; Bionote, Hwasung, Republic of Korea) according to the manufacturer’s recommendations for serum samples. The ELISA kit was initially validated for detecting anti-RRSV antibodies in serum or plasma from pigs but not for their detection in TOPF. The optical density (OD) was measured at 450 nm with the reference wavelength at 620 nm in an Infinite 200 PRO microplate reader (Tecan, Männedorf, Switzerland) immediately after the reaction was stopped. Signal-to-positive-control (S/P) ratios were calculated using the following equation: S/P = (OD_sample_ – OD_negative control mean_)/(OD_positive control mean_ – OD_negative control mean_). The sample was considered positive for PRRSV Ab if the S/P ratio was greater than or equal to 0.4 and was regarded as negative if the S/P ratio was less than 0.4.

To evaluate the pooling effect on the test results, samples were classified as low, moderately or highly positive (S/P ranges: 0.40–1.13, 1.14–1.86 and 1.87–2.60, respectively). For each range, four positive samples were randomly selected. Next, for pooling simulation, 10 μL aliquots of TOPF separated into low, moderately and highly positive were mixed with appropriate volumes of negative TOPF sample to simulate pools of 5, 10, 20, 40 and 80 samples containing 1 positive and 4, 9, 19, 39 and 79 negative samples, respectively. These pooled samples were then tested as described above.

### PRRSV genetic material detection and pooling simulation

This part of the study used 348 serum-and-TOPF pairs from male piglets and sera from 341 female piglets. Genetic material was isolated from 100 μL of each serum or TOPF sample following the manufacturer’s instructions using a Viral DNA/RNA kit (A&A Biotechnology, Gdańsk, Poland) and stored at –80°C until further processing. A commercial real-time reverse-transcription (RT)-PCR kit (ViroReal Kit PRRSV Type 1 & 2; Ingenetix, Vienna, Austria) was used for PRRSV RNA detection. The test detects strains of both PRRSV-1 and PRRSV-2. According to the manufacturer’s instructions, a sample was considered positive when the threshold cycle (Ct) was <45. A CFX96 PCR Detection System (Bio-Rad Laboratories, Hercules, CA, USA) was used for the analysis. To evaluate the pooling effect on the test results, samples were first classified as low, moderately or highly positive by Ct, sorting by the ranges ≥35, 25.01–34.99 and ≤25, respectively. Three positive samples were randomly selected from each range. Next, for pooling simulation, 10 μL aliquots of selected TOPF samples separated by Ct range were mixed with appropriate volumes of PRRSV-negative samples of TOPF to simulate pools of 10, 20, 40, 80, 160 and 320 samples containing 1 positive and 9, 19, 39, 79, 159 and 319 negative samples, respectively. Isolation of RNA isolation and a real-time RT-PCR were then performed on each diluted sample as described above.

### Statistical analyses

The Chi-square test of independence was used to determine whether there was a significant difference between the frequency of positive results obtained by the ELISA and the frequency of them as indicated by real-time RT-PCR in the male piglets, female piglets and TOPF with Bonferroni correction for pairwise comparisons. Differences in ELISA S/P values between results obtained from male piglet sera, female piglet sera and TOPF were tested by a nonparametric Kruskal–Wallis test followed by *post hoc* pairwise comparisons using Dunn’s test. The results from serum samples were used as a gold standard. Sensitivity (SE), specificity (SP), negative predictive value (NPV), positive predictive value (PPV) and Cohen’s kappa coefficient (κ) were calculated for the methods using TOPF instead of serum samples. Receiver operating curve (ROC) analysis was used to determine the optimal cut-off value for TOPF in both commercial tests. The cut-off that provided the best possible SE and SP (closer to the top left corner of the ROC) was selected. The analyses were performed using RStudio (version 4.1.2), except for the ROC, which was created using Statistica 13.3 (Tibco, Palo Alto, CA, USA). The significance level was α=0.05, and P-values < 0.05 were considered statistically significant.

## Results

### Detection of antibodies against PRRSV and pooling simulation

Of the male sera tested, 69.55% (217/312) were anti-PRRSV Ab positive. Among the corresponding TOPFs, 54.49% (170/312) of samples tested positive. Female piglet sera were positive for the antibody 74.52% of the time (196/263). Statistically significant differences were observed in the percentage of positive samples between boar sera and TOPF (P-value < 0.05) and between gilt sera and TOPF (P-value < 0.05) after using the manufacturer’s cut-off for both serum and TOPF samples. There were no significant differences in the percentages of positive samples obtained from boar sera and gilt sera (P-value = 0.187). The minimum, maximum and mean values of the samples’ S/P ratios are presented in Table 1. Statistically significant differences between the S/P values of boar sera and those of TOPF (P-value < 0.05) and between these values of gilt sera and of TOPF (P-value < 0.05) were found; however, there were no differences between the S/P values obtained from boar sera and those from gilt sera (P-value = 0.235).

**Table 1.** Minimum, maximum and mean sample-to-positive ratio (S/P) values in the sera of female and male piglets and in testicular-only processing fluid (TOPF)

Parameter	Females	Males	
	Serum S/P	Serum S/P	TOPF S/P
Min.	-0.018	-0.032	-0.030
Mean	1.412	1.279	0.694
Max.	3.500	3.220	2.610

**Fig. 1.**
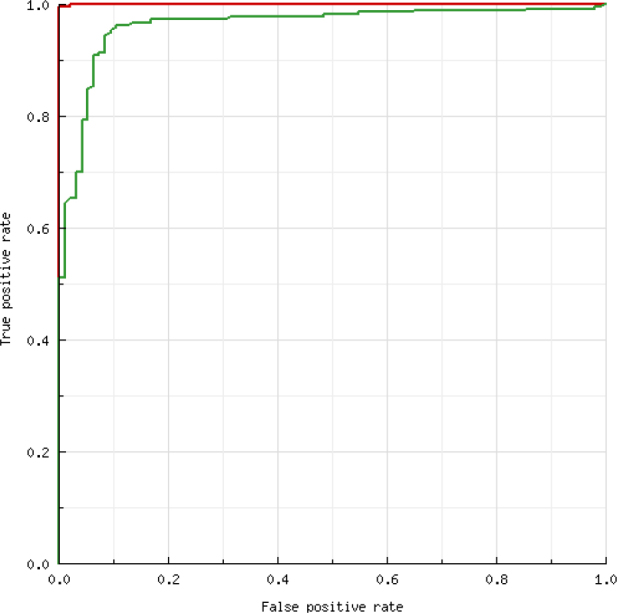
Receiver operating curve for interpreting the sample-to-positive-control (S/P) ELISA results in testicular-only processing fluid (TOPF). Red line – male serum S/P (reference); green line – TOPF S/P

The ROC analysis (Fig. 1) showed that the best accuracy (area under the curve (AUC): 96.34%) between TOPF and serum ELISA results occurred when the TOPF S/P threshold was ≥0.1. With this cut-off value, 219 (70.19%) TOPF samples were classified as positive for PRRSV-specific Ab, and no significant differences in the percentage of positive samples between boar sera and TOPF (P-value = 0.861) or between gilt sera and TOPF (P-value = 0.248) were observed. The SE, SP, NPV, PPV and κ calculated for TOPF using the cut-off recommended by the manufacturer of the ELISA assay and the ROC-calculated cut-off are shown in Table 2. The SE, NPV, and K values for TOPF improved significantly after using the new, lower cut-off point.

**Table 2.** Sensitivity (SE), specificity (SP), negative and positive predictive values (NPV and PPV), and Cohen’s kappa coefficient (κ) obtained for testicular-only processing fluid using the ELISA manufacturer’s and receiver operating curve (ROC)-calculated cut-off values

Parameter	ELISA cut-off	
	Manufacturer’s (S/P ≥ 0.4)	ROC-calculated (S/P ≥ 0.1)
SE (%)	76.96	86.47
SP (%)	96.84	96.31
NPV	0.65	0.95
PPV	0.98	0.91
κ	0.65	0.86

1 S/P – sample-to-positive-control ratio

Pooled TOPF samples were tested based on the ELISA manufacturer’s cut-off value to determine the maximum dilution to detect one positive sample in a pool of negatives. Anti-PRRSV antibodies were not detected at dilutions of 1 : 5 and higher in low-positive samples. Nevertheless, they were found in 20% (4/20) of these samples after applying the ROC-calculated cut-off. In moderately positive samples, 35% (7/20) were correctly classified as positive using the manufacturer’s cut-off and 90% were (18/20) using the calculated cut-off. In highly positive samples, 50% (10/20) and 100% (20/20) of samples were correctly classified as positive using the manufacturer’s and the ROC-calculated cut-offs, respectively. Detailed information regarding sample classification and S/P values after pooling one TOPF sample with an appropriate number of negative TOPF is presented in Table 3.

**Table 3.** The effect of pooling one low-, one moderately- and one highly-positive testicular-only processing fluid (TOPF) sample (classified by sample-to-positive-control ratio (S/P)) with 4, 9, 19, 39 and 79 negative TOPF samples

Dilution	Low-positive samples			Moderately-positive samples			Highly-positive samples		
	S/P^a^	Cut-off		S/P	Cut-off		S/P	Cut-off	
		Manufacturer’s S/P ≥ 0.4	Calculated S/P ≥ 0.1		Manufacturer’s S/P ≥ 0.4	Calculated S/P ≥ 0.1		Manufacturer’s S/P ≥ 0.4	Calculated S/P ≥ 0.1
Ndil	0.95			1.87			2.18		
1 : 5	0.35	0	1	0.99	1	1	1.32	1	1
1 : 10	0.08	0	0	0.51	1	1	0.99	1	1
1 : 20	0.05	0	0	0.22	0	1	0.54	1	1
1 : 40	0.04	0	0	0.17	0	1	0.24	0	1
1 : 80	0.02	0	0	0.12	0	1	0.13	0	1
Ndil	0.71			1.76			2.17		
1 : 5	0.13	0	1	0.96	1	1	1.16	1	1
1 : 10	0.08	0	0	0.48	1	1	1.01	1	1
1 : 20	0.04	0	0	0.26	0	1	0.52	1	1
1 : 40	0.02	0	0	0.16	0	1	0.31	0	1
1 : 80	0.01	0	0	0.11	0	1	0.22	0	1
Ndil	0.60			1.69			2.16		
1 : 5	0.11	0	1	0.81	1	1	1.11	1	1
1 : 10	0.07	0	0	0.41	1	1	0.62	1	1
1 : 20	0.03	0	0	0.21	0	1	0.38	0	1
1 : 40	0.01	0	0	0.1	0	1	0.25	0	1
1 : 80	0.00	0	0	0.08	0	0	0.16	0	1
Ndil	0.53			1.56			1.95		
1 : 5	0.14	0	1	0.75	1	1	1.01	1	1
1 : 10	0.07	0	0	0.24	0	1	0.65	1	1
1 : 20	0.03	0	0	0.19	0	1	0.15	0	1
1 : 40	0.00	0	0	0.12	0	1	0.2	0	1
1 : 80	0.00	0	0	0.09	0	0	0.14	0	1
Correctly classified									
1 : 5		0/4	4/4		4/4	4/4		4/4	4/4
1 : 10		0/4	0/4		3/4	4/4		4/4	4/4
1 : 20		0/4	0/4		0/4	4/4		2/4	4/4
1 : 40		0/4	0/4		0/4	4/4		0/4	4/4
1 : 80		0/4	0/4		0/4	2/4		0/4	4/4
Total		0/20	4/20		7/20	18/20		10/20	20/20

1 Ndil – not diluted

### PRRSV genetic material detection and pooling simulation

Of the 348 male sera tested, 74 (21.26%) were PRRSV RNA positive. Among the corresponding TOPFs, 53 (15.23%) samples tested positive. The quota of female serum samples tested was 341, and of them, 58 (17.00%) were positive for PRRSV RNA. No significant differences in the percentages of positive samples between boar sera, TOPF and gilt sera were found when using the manufacturer’s cut-off for serum and TOPF samples (P-value = 0.102). The minimum, maximum and mean Ct values are presented in Table 4.

**Table 4.** Minimum, maximum and mean threshold cycle (Ct) values in the serum of female and male piglets and in testicular-only processing fluid (TOPF)

Parameter	Females	Males	
	Serum Ct	Serum Ct	TOPF Ct
Min.	19.74	19.36	21.27
Mean	34.39	34.16	31.78
Max.	44.52	44.93	44.93

**Fig. 2.**
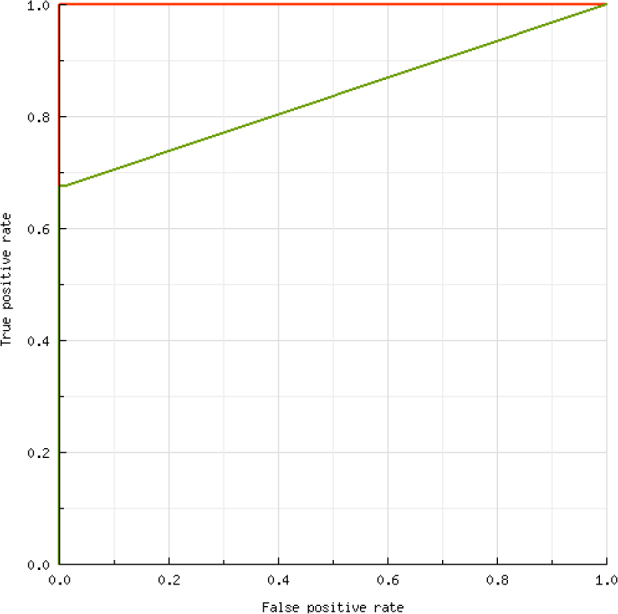
Receiver operating curve for interpreting threshold cycle (Ct) results in testicular-only processing fluid (TOPF). Red line – male serum Ct (reference); green line – TOPF Ct

The ROC analysis (Fig. 2) showed that the best accuracy (AUC: 83.61%) between TOPF and serum real-time RT-PCR results occurred when the Ct threshold for TOPF was established as <43.96. Using the ROC-calculated cut-off, 14.37% (50/348) of the TOPF samples were classified as positive for PRRSV RNA. After applying the new cut-off for TOPF samples, no significant differences in the percentage of positive samples between boar sera, TOPF and gilt sera were found (P-value = 0.054). The SE, SP, PPV, NPV and κ calculated for TOPF using the cut-off recommended by the manufacturer of the real-time RT-PCR assay and the ROC-calculated cut-off are shown in Table 5. The SP, PPV, and κ values for TOPF improved significantly after using the new, lower cut-off point. However, the SE stayed at the same level.

**Table 5.** Sensitivity (SE), specificity (SP), negative and positive predictive values (NPV and PPV), and Cohen’s kappa coefficient (κ) obtained in real-time reverse-transcription (RT)-PCR for testicular-only processing fluid using the manufacturer’s and the receiver operating curve (ROC)-calculated cut-off values

	Real-time RT-PCR cut-off	
Parameter	Manufacturer’s (Ct < 45)	ROC-calculated (Ct < 43.96)
SE (%)	67.57	67.57
SP (%)	98.91	100.00
NPV	0.94	0.92
PPV	0.92	1.00
κ	0.74	0.77

1 Ct – cycle threshold

Based on the cut-off value recommended by the manufacturer of the real-time RT-PCR test, TOPF pools were tested to determine the maximum dilution for detection to still be possible of one positive sample in a pool of negatives. In low-positive samples, viral RNA was detected in only 11.11% (2/18) of samples, regardless of the cut-off. In moderately positive samples, 72.22% (13/18) were classified as positive using the manufacturer’s cut-off, and 12/18 (66.66%) were using the ROC-calculated cut-off. All highly positive samples were classified correctly (100%; 18/18) regardless of the applied cut-off. Detailed information regarding sample classification and Ct values after pooling one positive TOPF sample with an appropriate number of negative TOPF samples is presented in Table 6.

**Table 6.** The effect of pooling one low-, one moderately- and one highly-positive testicular-only processing fluid (TOPF) sample with 9, 19, 39, 79, 159 and 319 negative TOPF samples

Dilution	Low-positive samples			Moderately-positive samples			Highly-positive samples		
	Ct^a^	Cut-off		Ct	Cut-off		Ct	Cut-off	
		Manufacturer’s Ct < 45	Calculated Ct < 43.96		Manufacturer’s Ct < 45	Calculated Ct < 43.96		Manufacturer’s Ct < 45	Calculated Ct < 43.96
Ndil	38.34	-	-	34.09	-	-	21.9	-	-
1 : 10	39.16	1	1	35.32	1	1	26.81	1	1
1 : 20	40.88	1	1	39.05	1	1	27.75	1	1
1 : 40	0	0	0	39.16	1	1	29.02	1	1
1 : 80	0	0	0	0	0	0	29.92		1
1 : 160	0	0	0	0	0	0	30.32	1	1
1 : 320	0	0	0	0	0	0	32.18	1	1
Ndil	43.7	-	-	34.16	-	-	22.62	-	-
1 : 10	0	0	0	35.24	1	1	25.08	1	1
1 : 20	0	0	0	43.00	1	1	25.45	1	1
1 : 40	0	0	0	43.66	1	1	27.08	1	1
1 : 80	0	0	0	44.71	1	0	27.85	1	1
1 : 160	0	0	0	0	0	0	28.04	1	1
1 : 320	0	0	0	0	0	0	29.43	1	1
Ndil	43.96	-	-	27.7	-	-	25.00	-	-
1 : 10	0	0	0	29.48	1	1	26.59	1	1
1 : 20	0	0	0	30.95	1	1	28.38	1	1
1 : 40	0	0	0	33.36	1	1	29.94	1	1
1 : 80	0	0	0	33.66	1	1	30.63	1	1
1 : 160	0	0	0	43.47	1	1	31.28	1	1
1 : 320	0	0	0	43.78	1	1	33.05	1	1
Correctly classified									
1 : 10		1/3	1/3		3/3	3/3		3/3	3/3
1 : 20		1/3	1/3		3/3	3/3		3/3	3/3
1 : 40		0/3	0/3		3/3	3/3		3/3	3/3
1 : 80		0/3	0/3		2/3	1/3		3/3	3/3
1 : 160		0/3	0/3		1/3	1/3		3/3	3/3
1 : 320		0/3	0/3		1/3	1/3		3/3	3/3
Total		2/18	2/18		13/18	12/18		18/18	18/18

1 Ct – threshold cycle; Ndil – not diluted

## Discussion

This study is focused on examining the potential of TOPF obtained only from piglet testicles collected during castration as an alternative matrix for a screening method for identifying the presence of anti-PRRSV Ab and PRRSV RNA in farrow-to-finish or farrow-to-weaning farms. In 2010, a European Declaration on alternatives to surgical castration aimed to end surgical castration by 2018 (4). However, most European countries have not yet met the target set by the declaration. Surgical castration of male piglets is still common on most commercial swine farms (1). There are available several alternatives to surgical castration which serve the same boar-taint-minimising purpose: slaughtering pigs before they reach sexual maturity (8), sperm sexing for the selection of female offspring, genetic selection for pigs with low levels of boar taint and immunocastration (23). Various concerns regarding alternative boar taint mitigation methods are raised because of the additional costs for farmers and uncertainties about consumer attitudes toward meat from pharmacologically castrated pigs. As a result, it is reasonable to assume that a complete ban on surgical castration, whether with or without anaesthesia, may not happen in the short term.

Piglet castration will likely continue to be permissible in Europe for the next few decades. In other major swine-producing countries such as China, the USA and Brazil, the issue of a castration ban has not yet been raised.

The commercial ELISA was initially dedicated to serum samples, and real-time RT-PCR tests dedicated to blood, saliva, semen, faeces, nasal secretions, milk and tissues were used. In order to investigate whether the results from PF obtained solely from boars (TOPF) differed from those obtained from gilt serum, serum taken from female piglets was also included in the analyses. Regarding serological analyses, a significant difference in the percentages of positive samples between piglet sera (boars and gilts) and TOPF was observed using the cut-off recommended by the manufacturer of the ELISA assay. It indicated that using an unmodified commercial ELISA protocol dedicated to sera to test TOPF led to an incorrect interpretation of the epidemiological situation in the herd. Our findings align with another study on TOPF, which utilised a commercial ELISA to identify antibodies against the hepatitis E virus in piglets. In this study, the agreement between serum and TOPF improved after recalculating the cut-off to a lower value (6).

Our results revealed that the concentration of antibodies specific to PRRSV in TOPF collected from 3–5-day-old boars was significantly lower than the concentration in their serum. Research on other biological fluids such as oral fluid or meat juice indicated that the antibody concentrations there were significantly lower than in sera (5, 21, 22). This phenomenon can be explained by the inclusion in PF or TOPF of other fluids (lymph and intracellular liquid) and blood which dilute the antibodies in the serum (6, 25, 28). Therefore, evaluating PF or TOPF with tests dedicated to serum without an experimental determination of the correct cut-off point may lead to an increased number of false-negative results. Our results suggested a considerably lower cut-off value is expected for TOPF when using an ELISA initially validated for serum samples.

Our study calculated the new cut-off value for TOPF samples to adapt such an ELISA test to be used with TOPF. After applying the ROC-calculated cut-off value for serological result interpretation, there were no statistically significant differences between the frequency of male piglet serum positive and negative results, the frequencies of female piglet serum results and their equivalents in TOPF results. Using the ROC-calculated cut-off value to interpret the TOPF results significantly improved the validation parameters of the test. The SE value increased from 76.95% to 89.47% and the NPV from 0.65 to 0.91. The two investigated matrices also had a higher agreement (κ = 0.65 *vs* 0.86) (Table 2). The validation parameters of the method in which TOPF was tested instead of serum in the ELISA test indicated that after applying a new cut-off value for TOPF, the probability of obtaining false-negative results decreased considerably without any significant increase in the frequency of false-positive results. Determining accurate cut-off values is essential for correctly interpreting the results when working with a new sample type in an ELISA. For instance, Henao-Diaz *et al*. (11) performed a study on oral fluid and discovered that changing the ELISA’s cut-off from S/P ≥ 0.4 to S/P ≥ 1.0 lowered diagnostic sensitivity slightly from 99.7% to 96.2%, but raised diagnostic specificity from 98.1% to 100% (11, 12).

There were no differences between sexes in the serum ELISA results or between results obtained from TOPF and from male and female serum. These results suggested that testing TOPF may be acceptable for assessing the health status of whole litters when only this material is analysed and no material from female piglets is tested. Validation of this claim requires further research, including on fluid obtained from PRRSV-positive gilts’ tails. However, considering the structure of the 3–5-day-old piglet’s tail, it is unjustified to expect it to yield a significant volume of fluid for serological testing, even after several defrosting cycles. Moreover, routine tail docking is not recommended, and more and more farms are abandoning this practice (9).

Our pooled-sample findings revealed that the final result depended on the initial concentration of antibodies in the single positive sample which was pooled with an appropriate number of negative samples. When the ROC-calculated cut-off value was utilised, the number of false-negative pooled samples decreased, and positive samples with higher dilution were still detected. A dilution of 1 : 5 or higher for low-positive samples resulted in a false-negative outcome when using the manufacturer’s cut-off. With the ROC-calculated cut-off, all low-positive samples were correctly classified in a dilution of 1 : 5. However, the higher dilutions were linked with false-negative results. In the moderately positive samples, antibodies were detected in all of them at the 1 : 5 dilution and three out of four samples at a 1 : 10 dilution using the manufacturer’s cut-off. After applying the new cut-off, all samples were correctly classified as positive at dilutions of up to 1 : 40 and two out of four were when at a dilution of 1 : 80. The overall percentage of correctly classified samples increased from 35% to 90%. Using the ROC-calculated cut-off value for result interpretation in the highly positive samples brought the results into full agreement with the results obtained from non-diluted samples, regardless of dilution (100% correctly classified pooled samples at dilutions up to 1:80). In contrast, when the original cut-off value was applied, 100% correctly classified pooled samples were noted up to 1 : 10. At 1 : 20 dilution, only two out of four samples were correctly classified, and higher dilutions gave negative results. It is important to note that the ELISA should be sensitive enough to detect at least one positive animal at a dilution of 1 : 20 when testing samples from farrowing pens containing as many as 20 piglets. After recalibrating the cut-off value, we successfully detected one positive sample commingled with 19 negative samples in all moderately and highly positive pools. With the new threshold applied, we accurately classified 42 diluted samples out of 60, compared to 17 samples out of the same 60 diluted samples using the manufacturer’s threshold. These results align with a similar study by Di Bartolo *et al*. (6) on detecting anti-hepatitis E antibodies in pooled TOPF samples.

Several studies highlighted the reliability of PF as a sample for molecular diagnosis of PRRS (15, 16, 25, 28). In our study, no significant differences in the percentage of positive samples were shown between piglet serum (from boars and gilts) and TOPF or between sexes using the manufacturer’s cut-off. The real-time RT-PCR assay performed on TOPF in our study showed very high SP (98.91%), moderate SE (67.57%) and high agreement between TOPF and male sera results (κ = 0.74). After applying the new cut-off (Ct < 44.96), we noted an increase in SP (98.91 *vs* 100%), and an increase in agreement (κ = 0.77) between these two matrices. The ROC-calculated cut-off point improved the method’s parameters (SP, PPV and κ) and validation when using TOPF as a diagnostic sample. Nevertheless, it is worth noting that the SE of the method was relatively low and did not change regardless of the selected cut-off. After implementing the new cut-off point, noticeable discrepancies in the percentages of positive samples between boar serum samples and TOPF were observed, indicating suboptimal validation parameters for the method. Conversely, no disparities were noted for gilts and TOPF, mainly because of the lower overall number of positive gilts. Not all true-positive cases may be detected when using TOPF as a diagnostic sample, especially those piglets with low positive Ct values in serum. The results of the real-time RT-PCR using TOPF for PRRSV genetic material detection should be interpreted cautiously. While a positive result is reliable, a negative result from PF carries a high risk of being a false negative. The utility of TOPF in PRRS diagnosis was recently assessed by Vilalta *et al*. (29) in 24 litters of piglets 10 weeks after a PRRSV outbreak. The results from TOPF, PF from tails only and udder wipes were compared with the results from piglet serum as a gold standard. Blood samples, tails and testicles from each piglet in the litter and an udder skin swab from the sow were collected at the time of castration and tail docking (3–5 days after birth). The optimal cut-off points in the real-time RT-PCR assay for classifying a sample as negative were calculated based on statistical analysis, and found to be ≥35 Ct for serum and ≥36 Ct for pooled samples (testes, tails and udder swabs). Adopting these cut-off points, the use of PF obtained from testes alone gave the best validation parameters and the highest SE (92%; SP = 82%, NPV = 90%, PPV = 85% and overall agreement = 87%). The SE of the assay on PF obtained from tails alone was moderate at 62% and from udder swabs was only 23%, with both matrices giving SP and PPV of 100% (29). Specificity, NPV and PPV were similar in our study. However, we obtained a lower SE. This could be attributed to different real-time RT-PCR assays having been employed in the cited research and in ours, to the aggregation of PF samples from litters as contrasted with the individual piglets tested in our study, or to possible cross-contamination between litters in our research.

In serum analysis conducted by López *et al*. (15), it was found that among 22 litters testing positive for PRRSV, 10 litters contained only a single PRRSV-positive piglet. Consequently, this led to the decision for the present research to take a methodological approach involving diluting one positive sample with multiple negative samples. However, there is a risk of obtaining false-negative results if the number of virus particles in the positive sample is low. López *et al*. (17) stated that including two or more viraemic piglets in a sample increased the likelihood of detecting PRRSV. Combining samples from multiple litters can improve the chances of finding PRRSV-positive piglets. However, we must be cautious when diluting the samples with TOPF from numerous non-viraemic piglets. Our study found that using TOPF as a diagnostic sample in a real-time RT-PCR commercial kit resulted in suboptimal validation parameters. At dilutions up to 1 : 20, only one out of three low-positive samples was correctly classified as positive, representing 11.11% of all correctly classified samples in this group, regardless of the cut-off point used. Among the moderately positive samples, only one of the three selected samples with the highest Ct was correctly classified at dilutions up to 1 : 320, when both cut-off points were used for results interpretation. Overall, even fewer samples were correctly classified as positive after using the new ROC-cut-off in this group (72.22% *vs*. 66.66%). Interestingly, despite dilution with 319 negative samples, all highly positive samples in our study were still correctly identified with both cut-off points, indicating high accuracy. However, it is important to note that this high accuracy depends on having a high Ct value in the positive TOPF sample. Similar results were observed in a study by López *et al*. (16). They found that the probability of correctly classifying a sample as positive was 95% in a 323-piglet sample containing 28 PRRSV-negative litters and 1 PRRSV-positive litter. According to the regression analysis model proposed by López *et al*. (16) for the serially diluted processing fluid sample, limiting the processing fluid sampling to 30 litters or fewer (around 320 piglets) was expected to provide 95% confidence in detecting PRRSV RNA, even when the prevalence within piglets was close to zero.

We also noted three false-positive results in our study, where a high Ct was obtained in the PF samples but no Ct was measured in the corresponding serum samples. It is important to consider that the virus may be transmitted through contaminated hands or equipment used during castration (18). Collecting blood into a sterile test tube differs from the lengthier and more involved castration procedure for piglets; the longer procedure could cause the contamination of TOPF samples obtained from piglets castrated by veterinarians who had had previous contact with an infected animal. Veterinarians should be mindful of changing gloves and other equipment after each piglet, although this can be challenging in field conditions. Doing so can help prevent false-positive results and cross-contamination, particularly between individual litters or batches.

Our research has shown that TOPF samples can be valuable for diagnosing PRRS and monitoring PRRSV in piglets (boars and gilts). Testing for anti-PRRSV Ab and PRRSV RNA in TOPF samples provides a quick, reliable and economical way to assess the status of PRRSV on pig farms without causing stress or harm to the animals. However, it is important to establish the appropriate cut-off point or dilution for this type of sample and the specific ELISA test to maximise the chances of obtaining the optimal validation parameters. Another limitation is that pooled-sample results from ELISAs and real-time RT-PCRs may not accurately indicate the actual PRRSV status of the herd, especially when they contain TOPF samples with low levels of Ab or viral load. Using an appropriate cut-off point or sample dilution when testing pooled samples in commercial ELISA kits is advisable to reduce the probability of false negatives. The validation parameters of the method using TOPF for molecular testing are not satisfactory because of the low sensitivity, regardless of the cut-off point used. Therefore, the results of TOPF testing for the presence of PRRSV genetic material should be interpreted with caution, and it is crucial to be aware of the potential for false-negative results, especially from positive individuals with high Ct in serum, as well as to be aware of the possibility of false-positive results from cross-contamination during castration. Nevertheless, the result concordance between TOPF and samples collected from females indicates that TOPF could be used for diagnostic purposes for a whole litter.

## Conclusion

Testicular-only processing fluid samples can be a valuable tool for laboratory PRRS diagnosis in piglets. However, it is important to be aware of the potential for false-negative results.
